# Standardized Sensitivity Analysis in BCA: An Education Case Study

**DOI:** 10.1017/bca.2019.5

**Published:** 2019-03-06

**Authors:** Elina Pradhan, Dean T. Jamison

**Affiliations:** 1 Elina Pradhan, The World Bank Group, Health, Nutrition and Population Global Practice, Washington, DC 20433, USA; 2 Dean T. Jamison: University of California, San Francisco, CA 94143, USA

**Keywords:** education and human capital, health, D61, I1, I15, I18, I26, O2

## Abstract

Benefit-cost analyses of education policies in low- and middle-income countries have historically used the effect of education on future wages to estimate benefits. Strong evidence also points to female education reducing both the under-five mortality rates of their children and adult mortality rates. A more complete analysis would thus add the value of mortality risk reduction to wage increases. This paper estimates how net benefits and benefit-cost ratios respond to the values used to estimate education’s mortality-reducing impact including variation in these estimates. We utilize a ‘standardized sensitivity analysis’ to generate a range of valuations of education’s impact on mortality risks. We include alternative ways of adjusting these values for income and age differences. Our analysis is for one additional year of schooling in lower-middle-income countries, incremental to the current mean. Our analysis shows a range of benefit-cost ratios ranging from 3.2 to 6.7, and net benefits ranging from $2,800 to $7,300 per student. Benefits from mortality risk reductions account for 40% to 70% of the overall benefits depending on the scenario. Thus, accounting for changes in mortality risks in addition to wage increases noticeably enhances the value of already attractive education investments.

## 1 Introduction

In 2016 the International Commission on Financing Global Education Opportunity published its report, *The Learning Generation: Investing in Education for a Changing World* (The International Commission on Financing Global Education Opportunity, 2016). The Commission, chaired by former UK Prime Minister Gordon Brown, utilized benefit-cost analyses (BCAs) to underpin its conclusion that a major acceleration of education is now warranted. Standard BCA methods were used to show large incremental benefits as compared to incremental costs, weighing costs of schooling, including opportunity costs of student time, against estimates of health benefits and education-related wage gain over the individual’s working lifetime (Psacharopoulos et al., 2017).

The Commission pointed to a diverse and substantial literature relating higher levels of education to lower levels of premature mortality, and to improved health more generally (Filmer & Pritchett, 1999; Gakidou et al., 2010; Baird et al., 2012; Kuruvilla et al., 2014; Wang et al., 2014; Jamison et al., 2016). The Commission requested that a team (assembled by SEEK Development in Berlin) utilize this literature to extend standard approaches to BCA in education to include explicit valuation of reasonable estimates of the impact of education on mortality. The Commission then featured the results of that assessment in the executive summary of their report. Pradhan et al. (2018) report those findings – and the methods on which they are based – in detail.

A notable difficulty facing the Pradhan et al. (2018) team concerned how to value small changes in mortality risk using monetary measures (derived from the revealed or stated preferences of individuals and conventionally reported as the value per statistical life, VSL). By now, a substantial empirical literature reports findings from different approaches to estimate monetary value of mortality risk reductions in low- and middle-income countries (Narain & Sall, 2016; Viscusi & Masterman, 2017*a*,*b*). That said, huge variation remains in how one might plausibly apply existing results in a broad range of contexts, particularly in low-income or high-mortality environments. The Pradhan et al. study selected reasonable values of parameters from the literature, but other analysts could have plausibly chosen different parameters. This indeterminateness limits the utility of most BCAs for two reasons:

(i) It can be hard to judge how sensitive the study’s findings are to parameters chosen; and
(ii) Individual BCA results cannot be easily compared across sectors and over time to generate a solid corpus of comparable findings. (This problem applies equally to the valuation of mortality risk reductions when assessing the economic burden of diseases or risk factors.)

Analysts from several governmental and international organizations and researchers have proposed varying approaches for increasing standardization. Chang et al. (2018) provide a brief account of some options that have been considered. The Bill & Melinda Gates Foundation has funded development of a BCA reference case (Robinson et al., 2019*a*) to address these problems in a way broadly consistent with a cost-effectiveness reference case that the Gates Foundation had previously funded (Wilkinson et al., 2016). This education case study was prepared to support and illustrate the development of the BCA reference case.

One suggested approach to improving credibility and comparability was that of developing a ‘standardized sensitivity analysis’ (SSA) to be reported routinely within BCAs. The analyst might choose to highlight a particular value from the SSA as the best estimate in the analysis undertaken. Or, for any of a variety of reasons, the analyst might choose to headline a different approach but to undertake the SSA as well. The SSA would both enable judgement on the robustness of the findings of the analysis and enable comparison of those findings with an accumulating literature. The value of developing a reference case lies in enabling this comparability.

Our purpose in this case study is to illustrate how SSAs might reasonably be done. The analysis assesses the costs and benefits of education investments in lower-middle-income countries (LMCs)^1^ from a perspective where we estimate the economic returns to education from the value of reductions in under-five and adult mortality risks in addition to any increases in earnings. We develop a case study that starts with the Pradhan et al. (2018) findings, but then reanalyse those findings using estimates of the value of mortality risk reduction that vary by income and age. An important output from Pradhan et al. (2018) was assessment of the benefits of *quality* of education as well as years of schooling. Not surprisingly, quality proves important. For simplicity, this paper illustrates the results of the SSA using only the estimated effects of years of schooling on mortality risks.

Before proceeding to the main body of the paper, we discuss how the purposes of a BCA affect the way(s) it might most usefully be reported. Perhaps the most important and typical use of BCA is to assist with determinations of how (and whether) specific major projects or regulations should be undertaken. The present value of the net benefits (PVNBs) of options considered provides the key input to decision makers, and its calculation and reporting is a central task for the analyst.

Less often – but still importantly – the BCA’s purpose is to contribute to an evolving literature about what works (and what does not) and in general to convey how attractive certain classes of investments tend to be. The analysis reported in this paper falls squarely in this latter, evaluative category. The intention is to add to a literature that provides conclusions relevant to a broad range of contexts. Benefit-cost ratios (BCRs) can be understood as relevant to many scales and contexts thereby providing useful (if only broad brush) policy guidance. Both PVNB and the BCR require an exogenously-given discounting procedure and may be (usually are) quite sensitive to choice of procedure as we shall see later in this paper.

The economics of education literature borrows from the finance literature in generally reporting the results of its BCAs as internal rates of return (IRRs). The IRR is that value of a constant discount rate that solves the equation equilibrating the time streams of benefits and costs. (A problem is that this equation may have multiple solutions although this does not appear to be a problem in practice in the education literature.) An advantage is that the discount rate is an output from rather than an input to the IRR and, like the BCR, it shows relative attractiveness independent of scale enabling comparison of economic attractiveness across studies. However, like the BCR but unlike PVNB, the IRR gives no indication of absolute attractiveness.

A standardized sensitivity analysis serves the purpose of accumulating knowledge across studies. Hence in this paper we explore the SSA by way of BCRs and – since the topic is education (and we seek to relate to that literature) – we also report IRRs as well as PVNB.

The case study proceeds as follows. The next section describes our approach to the SSA. Following the SSA methods section, section C describes the BCA undertaken for the Brown Commission (Pradhan et al., 2018); in particular the analysis estimating BCR, PVNB and IRR in LMCs for one year of schooling incremental to the current mean of 7 years. Section D presents results of the SSA, and section E discusses the findings and study limitations.

## 2 Methods

Our methods begin with those standard in the economics of education literature which construct time streams over individual lifetimes of education costs and benefits, where benefits are the estimated earnings increases associated with the increment in education being considered (here one-year incremental to 7 years). We add to that the estimated value of mortality reduction in the cohort associated with that one-year increment in education. The methods discussion that follows concerns how we translate an estimated reduction in numbers of deaths into a dollar benefit. The short answer is to multiply the number of deaths estimated to be averted by the ‘value of a statistical life’ or VSL. Operationalizing that short answer in the current context is the subject of the next few pages.

We vary the value of mortality risk reductions on income and age dimensions. The first dimension is concerned with how the analysis treats variation across country income levels, based on how the ratio of the value of a unit of mortality risk reduction to gross national income (GNI) per capita (purchasing power parity (PPP) adjusted) varies with income. We report our SSA using three alternatives recommended by Robinson et al. (2019*b*) – (i) the ratio of VSL-to-GNI per capita remains constant at 160, using U.S. values as a starting point and an income elasticity of 1.0; (ii) the ratio of VSL-to-GNI per capita remains constant at 100, using OECD values as the starting point and an income elasticity of 1.0; and (iii) the ratio of VSL-to-GNI per capita has an income elasticity of 1.5, using the U.S. ratio of 160 as a starting point.

The first two income variants are included to facilitate comparisons with existing research that uses these alternative approaches. The third option, which Robinson et al. (2019*b*) propose as the featured variant, reflects the expectation that the VSL-to-GNI per capita ratio will be smaller in LMCs than in a high-income country (the United States in this case), given the need to preserve more limited income for essential expenditures. These three variants demonstrate uncertainties in the VSL estimates, given that few empirical studies have been conducted in LMCs.

Application of the first two variants is straightforward. For the third variant, we calculate the ratio of VSL-to-GNI per capita starting with the formula from Hammitt and Robinson (2011): $VSL=VSL_{t}\times {\left(\frac{GNIpercapita}{GNIpercapita_{r}}\right)}^{elasticity}$, where *VSL_r_* is the recommended reference value, in this case from the United States.

We reformulate this as the VSL-to-GNI per capita ratio (VSLR) = VSL/GNI per capita where,

(1) $$(\frac{VSL_{LMCs}}{GNIpercapita_{LMCs}})=\left(\frac{VSL_{US}}{GNIpercapitaUS}\right)\times {\left(\frac{GNIpercapita_{LMCs}}{GNIpercapita_{US}}\right)}^{elasticity-1}$$

Here, *GNI per capita_US_* = $57,900, *GNI per capita_LMCs_* = $6,430, *VSL_US_* = 160 × $57,900, and elasticity = 1.5.

Therefore, *VSLR_LMCs_* = 54.

The second dimension is concerned with how the analysis values mortality risk reductions at different ages, and we select the two alternative approaches discussed in Robinson et al. (2019*b*): (i) the value does not vary with age; and (ii) the value is proportional to remaining life expectancy. The second age variant suggests estimating a constant value per statistical life year (VSLY) based on each of the population-average VSLs estimated using the approaches referenced above. The VSL is divided by undiscounted remaining life expectancy at midpoint of the approximate life expectancy at birth for the region of interest (age 35 in this case). The resulting VSLY is then multiplied by change in the future life expectancy of the population affected. Note that in the first scenario where the VSL does not vary with age, VSL is a population-average estimate that is applied to all beneficiaries of the intervention. The empirical research upon which these estimates are based relies largely on samples of working-age adults. Whether these values are appropriate for younger or older populations is uncertain (Hammitt, 2007) even in high-income countries; little is known about the relationship of the VSL to age or life expectancy in LMCs. As Robinson et al. (2019*b*) note, although not robustly supported by available research, the second age variant of assuming a constant VSLY provides a rough proxy for the effects of age and life expectancy.

Table 1 summarizes these approaches, including three income variants combined with two age variants.

**Table 1:** The six outputs of a Standardized Sensitivity Analysis for LMCs^a^.

Variation of value with GNI per capita	Variation of value with age	
	1. None (Population-average VSL)	2. Proportional to remaining life expectancy (Age-adjusted VSL)^b^
1. Constant VSL-to-GNI per capita ratio (D160)	VSL .1, 1) = 160 × $6,430 = $1,100,000	$VSL(a)\text{ (1,2) = \$1,100,000 }\times \;\frac{L(a)}{L(35)}$
2. Constant VSL-to-GNI per capita ratio (D100)	VSL (2, 1) = 100 × $6,430 = $643,000	$VSL(a)\text{ (2,2) = \$643,000 }\times \;\frac{L(a)}{L(35)}$
3. Varying VSL-to-GNI per capita ratio with income elasticity = 1.5	VSL (3, 1) = 54 × $6,430 = $347,000	$VSL(a)\text{ (3,2) = \$347,000 }\times \;\frac{L(a)}{L(35)}$

^a^Our case study is for LMCs as a group. Their per capita GNI in 2015 was $6,430 in PPP (current international $) (WDI 2017). With the income per capita of the case study region, we can fill in the relevant values of column 1 of Table 1 (since values in column 1 are independent of the age distribution of mortality changes). The outputs are the value of the benefits of the intervention as specified in (2) below. The SSA scenarios are based on recommendations from Robinson et al. (2019*b*).

^b^*L*(*a*) is life expectancy at age a, and L(35) is remaining life expectancy at midpoint of the approximate life expectancy at birth for LMCs (age 35 in this case).

In addition to the SSA comparisons above, we perform a sensitivity analysis across discount rates, and for SSA scenario (3,1), we evaluate the sensitivity of the BCA estimates across a wider range of income elasticities.

More generally, if *δ(a)* deaths/year is the annual age-specific benefit of the intervention in mortality risk reduction (at age *a*), *y* = income, and *η(a)* is the age distribution of the population to which the intervention applies, then:

(2) $$\text{Value of intervention }\left(\text{in }\$/\text{year}\right)=\int_{0}^{\infty }\delta \left(a\right)VSL\left(y,a\right)\eta \left(a\right)da.$$

## 3 Cost-benefit analysis of additional schooling

Analyses prior to Pradhan et al. (2018) have estimated the returns to education using household and labour market survey data, and mainly focus on the private and ‘social’ returns to years of schooling in terms of earnings. IRR for education investments is the discount rate at which net present benefits of the education investments are zero. The ‘social’ IRR incorporates the full cost of schooling and pretax earnings, whereas private IRR estimates assume after-tax earnings and that the cost of schooling is borne by the government, and the only cost of schooling to the individual is the opportunity cost of time associated with attending school. Both these estimates traditionally only consider the wage benefits of increased schooling, with the private returns considering after-tax wages.

Pradhan et al. (2018) expand on the traditional approach to BCAs by including health gains to increased schooling in addition to the earnings return, and estimate the PVNBs, IRRs and BCRs of investing US$1 in education in low-, lower-middle-, and upper-middle-income countries from a societal perspective. This case study presents a proposed set of standard sensitivity analysis on BCRs of an additional year of schooling in LMCs. We also updated our data sources for our analysis as compared to Pradhan et al. (2018), as tabulated in the Appendix.

The methods for BCA of additional schooling are briefly summarized below:

### 3.1 Benefits of additional schooling

Pradhan et al. (2018) use a hierarchical linear model to estimate the impact of increased female schooling on under-five mortality, adult male mortality and adult female mortality controlling for technological progress (proxied by time-period categories) and income. Further, we allow the impact of technological progress to vary every five years, hence allowing a country-specific impact of technological progress on health. We replicate the different approaches to estimating the macro-level impact of education on health found in the literature, and choose the model with the most robust controls, resulting in an estimate that is a lower bound of the range used in the same paper and in this case study.

From these regression results, we estimate the level of reductions in (1) adult female mortality, (2) adult male mortality, and (3) under-five mortality (from mother’s education) resulting from one more year of female schooling as was done in Pradhan et al. (2018). Since the average years of schooling in LMCs is seven years, the BCR calculations estimate the benefits and costs of increasing schooling from seven years on average, to eight years per pupil.

The mortality reductions are then valued using base VSL-to-GNI per capita ratio of 160, another ratio of 100, and discount rates including 1%, 5% and 8.4% with the ratio of 160. The main analysis discounts future costs and benefits at 3%. We combine the expected health value with the earning benefit of increased schooling using smoothed age-earnings profiles received from Psacharopoulos et al. (2017). The education to earnings link accounts for increased productivity, but it could also account for increased life expectancy or reduced morbidity which would also improve lifetime earnings.

Note that the impact of increasing schooling on earnings is estimated for both male and female pupils – we use the regional average increase in earnings by increasing one year of secondary schooling. The mortality effect of male education appears to be minimal. The costs in our BCA include costs for educating both male and female students.

### 3.2 Costs of additional schooling

The direct cost data was provided by International Commission on Financing Global Education Opportunity, which is the cost of teacher time, facilities rent and consumable items such as textbooks, and the opportunity cost of student time was derived from the age-earnings profile. The opportunity cost is the earnings forgone by the additional year of schooling, such that the earnings for the age of entry for additional year of schooling is negative. The direct cost of schooling is only incurred in the additional year; it is zero for ages higher than the age at which the additional schooling occurs. Similar to direct costs, the opportunity cost of schooling at ages higher than the age of additional year of schooling is also zero.

### 3.3 Health-inclusive benefits and costs of additional schooling

The health-inclusive IRR *(hPVNR(r_h_))* is the value of annual IRR *(r_h_)* such that the health-inclusive PVNB of an additional year of schooling is zero. The education literature typically reports the results of BCAs as IRRs for two reasons. One is to avoid selecting a discount rate from the sea of alternatives. The second is that, like the BCR and unlike the PVNBs – many believe the IRR provides an easily understood and cross-intervention compatible metric of attractiveness. We also report PVNBs, because only reporting a ratio can obscure the relative magnitude of the effects.

As described above, we consider annual direct costs *c*_1_*(a)* and opportunity costs of schooling *c*_2_*(a)*, and the health (mortality risk reduction) benefits *hv(a)* and earnings benefit *ev(a)* when estimating the rate of return. Equation (3) gives the net present value of health-inclusive costs and benefits of an additional year of schooling for ages A through 65. Age A is the age of entry for the additional year of schooling at the mean years of schooling (7th grade), which is at 14 years for LMCs.

(3) $$hPVNR\left(r_{h}\right)=\overset{65}{\underset{a=A}{\Sigma }}\frac{ev\left(a\right)+hv\left(a\right)-c_{1}\left(a\right)-c_{2}\left(a\right)}{(1+r_{h}{)}^{a-A}}.$$

Hence, the health-inclusive IRR is the value of *r_h_* where the net present value *(hPVNR(r_h_))* equals zero. Across a range of reasonable values, inclusion of the benefits from reduced mortality increases IRRs for education by over 40%. For example, in the specific calculation reported in Pradhan et al. (2018) for LMCs, the estimated IRR increased from 7.0% to 9.3% when the value of mortality reduction was included.

Similarly, the BCR is estimated by applying an annual discount rate (*r*) to all costs and benefits. For annual costs and benefits described above, equation (4) shows the health-inclusive BCR (hBCR) and equation (5) the health-inclusive PVNB of one additional year of schooling.

(4) $$hBCR\left(r\right)=\frac{\overset{65}{\underset{a=A}{\Sigma }}[{e\left(a\right)\left(1+r\right)}^{A-a}+{h\left(a\right)\left(1+r\right)}^{A-a}]}{\overset{65}{\underset{a=A}{\Sigma }}[{c_{1}\left(a\right)\left(1+r\right)}^{A-a}+{c_{2}\left(a\right)\left(1+r\right)}^{A-a}]}$$

(5) $$hPVNB\left(r\right)=\overset{65}{\underset{a=A}{\Sigma }}[\{e\left(a\right)+h\left(a\right)-c_{1}\left(a\right)-c_{2}\left(a\right)\}{\left(1+r\right)}^{A-a}].$$

The details of the estimation process of benefit and cost streams are explained in supplement section A, and supplement section D details the age- and income-adjusted benefit streams used for the SSA.

## 4 Results of BCA for education: standardized sensitivity analysis

Table 2a shows the present dollar value of reduction in mortality risk per student due to an additional year of schooling in LMCs, with varying assumptions of the dependency of the VSL on (i) the base VSL-to-GNI per capita estimate and the income elasticity, and (ii) age of the population groups affected; table 2b shows the BCRs, IRRs and PVNBs for the same. Note that our models assume that the health benefits accrue only to females who receive additional schooling but that the wage benefits accrue to both males and females. Hence, the estimate of the dollar value of health benefits is a weighted average with the weight depending on the fraction of the educated cohort that is female. The calculations assume the cohort is 50 percent female.

**Table 2a:** Present dollar value of mortality reduction benefits and increased earnings per student, in 2015 US$^a^.

Income variants	Present dollar value of mortality reduction benefits		Present dollar value of increased earnings
	Age variants		
	1. None (Population-average VSL)	2. Proportional to remaining life expectancy (Age-adjusted VSL)	
1. VSL-to-GNI per capita ratio = 160	$4,900	$6,100	$2,500
2. VSL-to-GNI per capita ratio = 100	$3,000	$3,800	
3. VSL income elastic^b^	$1,600	$2,100	

^a^Intervention is one additional year at the current mean of 7 years. Future benefits discounted at the rate of 3% per year.

^b^Since income is $6,430 per year, VSLR is 54 given an income elasticity of 1.5.

**Table 2b:** BCA for an additional year of education in LMCs: A standardized sensitivity analysis^a^.

	Age variants					
	1. None (Population-average VSL)			2. Proportional to remaining life expectancy (Age-adjusted VSL)		
Income variants	BCR^b^	IRR	PVNB^d^	BCR^b^	IRR	PVNB^d^
1. VSL-to-GNI per capita ratio = 160	5.8	14%	$6,100	6.7	18%	$7,300
2. VSL-to-GNI per capita ratio = 100	4.3	11%	$4,200	4.9	14%	$5,000
3. VSL income elastic^c^	3.2	9%	$2,800	3.6	10%	$3,300

^a^Intervention is one additional year at the current mean of 7 years.

^b^BCRs calculated using a discount rate of 3% per year.

^c^Since income is $6,430 per year, VSL-to-GNI per capita ratio is 54 given an income elasticity of 1.5.

^d^PVNB in 2015 US$.

We find that the present dollar value of mortality risk reduction is 180% higher if we assume the VSL-to-GNI per capita ratio is constant at 160 (an income elasticity of 1.0), as compared to when we use this U.S. ratio as the starting point and assume that VSL is income-elastic (with an elasticity of 1.5). In the first two income variant scenarios where the VSL-to-GNI per capita ratios are 160 and 100, the present dollar value of mortality reduction benefits exceeds the value of increased earnings. Additionally, if we assume that the value of mortality benefits is age-dependent, then the present dollar value of mortality reduction benefits increases by about 25%, as compared to assuming that mortality reduction benefits are independent of age.

Our results show that using the age-adjusted VSLs rather than the population-average VSLs yields higher values of mortality reduction benefits, BCRs and IRRs, because the benefits accrue to those who are younger than the population average. Every dollar invested in schooling in LMCs would return $5.8 in earnings and reductions in under-five and adult mortality when not adjusting the VSL for age (and when assuming the ratio of VSL-to-GNI per capita is constant at 160). However, adjusting the VSLs for years of life lost, the BCR is 17% higher, at $6.7 in benefits accrued per dollar spent on schooling. We also find that adjusting VSL for income elasticity of 1.5 yields lower IRRs, BCRs and PVNB for LMCs because the income of the reference country is about 8 times higher than the GNI per capita of LMCs at $6,430.

**Figure 1:**
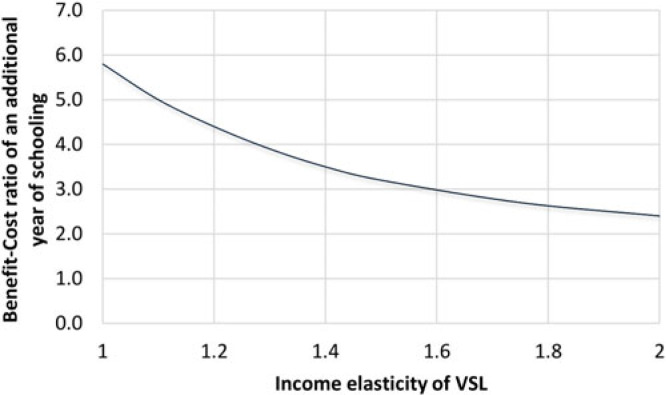
Relationship between benefit-cost ratio of an additional year of schooling and income elasticity of VSL. *Note*: Intervention is one additional year at the current mean of 7 years. Benefit-cost ratios are estimated for age variant 1, or the case in which VSL does not depend on age. VSL = Value of a Statistical Life.

Figure 1 presents the sensitivity of BCRs to income elasticity of VSL, using the U.S. value of 160 as the base estimate for the VSL-to-GNI per capita ratio. This scenario assumes that VSL is independent of age, and the future benefits and costs are discounted at the rate of 3%. BCRs and IRRs for LMCs decrease when we assume VSL is income elastic as the income per capita of the reference country (United States) is higher than the income per capita of LMCs. We find that the BCRs range from 2.4 to 5.8 when changing the income elasticity of VSL from 2.0 to 1.0. Note that elasticity of 2.0 is quite high given discussions and recommendations from Robinson et al. (2019*b*) – given that the two elasticities recommended are 1.0 and 1.5 with 1.5 recommended as the main one, we simply wanted to estimate the BCRs on the high end as well.

We also test the sensitivity of our results to four discount rates. The first is a rate of 3 percent, consistent with the International Decision Support Initiative (iDSI) Reference Case (Wilkinson et al., 2016) recommendations as well as common practices. We also report our results for discount rate approximately twice the GDP per capita growth rate in LMCs (Table 3), motivated by the Ramsey rule^2^ (see Claxton et al., 2019). The BCR with a discount rate twice the per capita growth rate seen in LMCs (*r* = 8.4%) is three times lower than at the standard rate of 3%. Additionally, the lower discount rate of 1% might be of particular importance because we assume that for each student affected, the increase in earnings accrues from age 14 (average age of entry for the additional year of schooling) to 65 (retirement age), and the mortality risk reduction accrues over 51 years. Comparing the age-unadjusted, income variant 1 scenario across the discount rate of 1% with the standard rate of 3%, we find that the lower discount rate yields a substantially higher BCR at $9.4 in benefits for every dollar spent in schooling in LMCs – this estimate is 62% higher than the BCR estimated at 3%.

**Table 3:** PVNBs and BCRs for an additional year of schooling: Sensitivity analysis on discount rates^a^.

Income variants	PVNBs^b^			BCRs		
	Discount rate (r)^c^			Discount rate (r)^c^		
	1%	5%	8.4%^e^	1%	5%	8.4%^e^
1. VSLR = 160	$10,700	$3,500	$1,400	9.4	3.8	2.1
2. VSLR = 100	$7,900	$2,300	$700	7.2	2.8	1.5
3. VSL income elastic^d^	$5,700	$1,300	$100	5.5	2.0	1.1

^a^ Intervention is one additional year at the current mean of 7 years.

^b^ PVNB in 2015 US$.

^c^ *r* = annual discount rate.

^d^ Since income is $6,430 per year, VSLR is 54 given an income elasticity of 1.5.

^e^ Since 2015 GDP per capita growth rate for LMCs was 4.2%, this discount rate reflects twice the near-term growth rate.

## 5 Discussion

BCAs in education weigh future education-associated wage increases against the current costs of providing schooling and the opportunity costs of students’ time. Female education is reliably estimated to reduce both child and adult mortality rates, and estimates reported in this paper, although at the bottom of the published range, prove quantitatively significant. The paper explores a standardized approach to valuing those mortality reductions, then adds those values to wage benefits to generate more robust BCAs for education investments. Before discussing those SSAs, it is worth making our subject and conclusions concrete. The investment we assess is to add one year to the current average of seven years of education in LMCs (as defined by the World Bank). For 10,000 students, a reasonable estimate of costs is $13 million (in 2015 US$). This increase in (female) education would, over the next half century, result in an estimated reduction in 31 deaths under age five and 64 deaths between ages 15 and 60. It would result in an (undiscounted) increase in earnings of about $60 million assuming no trend increase in wages (or, discounted at 3% per year, a $25 million increase). This is the starting point for the SSA.

This case study applies a standard sensitivity analysis that values the impact of increased education on longevity and earnings. We find that the mortality risk reduction is a sizeable fraction of the benefits of education – perhaps 40–70% of the total including increased earnings. BCRs are highly sensitive to the discount rates used and the VSL assumptions. For example, with a VSL-to-GNI per capita ratio of 160 and income elasticity 1.5, BCRs at a 1% discount rate are twice as high as the estimates at 3% discount rate, and five times higher than the estimates at 8.4%. Those caveats noted, we conclude that investing in an additional year of schooling is likely to have a BCR of greater than 2. Regardless of the SSA scenario, at least a third of the benefits result from the estimated effect of female education on the adult and under-five mortality risks.

We find that every dollar invested in schooling in LMCs would return between $3.2 to $6.7 in benefits in increased earnings and mortality risk reductions, testing the sensitivity of the BCR to differing assumptions regarding the value of mortality risk reductions and its variation by age. Zooming in on the health returns of increased schooling, the sensitivity analysis finds that the present dollar value of mortality risk reduction benefits of increasing one year of schooling ranges from $1,600 to $6,100 per student.

Another sensitivity analysis we performed was around the income elasticity of VSL. We find that the BCR decreases by 58% when we start with the U.S. VSL-to-GNI per capita ratio and change income elasticity of VSL from 1 to 2. Additionally, we find that BCR estimates are sensitive to the discount rates used – in one scenario of BCR estimation, changing the discount rate from the standard 3% to 1% results in a 62% higher BCR.

This case study estimates the direct longevity and earnings effects of increasing schooling. However, income affects health, and education affects both health and income. Further work needs to be done to estimate these indirect effects of education on health as mediated through income. Our statistical model in effect holds income constant while estimating the magnitude of education’s effect on mortality. It is this (conservative) estimate that we report. We do know, in addition, that education has an important effect on income and income has a (modest) effect on mortality. Further research should quantify all channels of education’s effects on mortality. When that research agenda has been completed, we believe our estimate will be shown to have been an underestimate, albeit a modest one. One additional question is the extent to which the VSL includes the effects of mortality risk reductions on earnings. Presumably, VSL reflects individuals’ current earnings expectations, not their expectations conditional on increased education. The interaction of these two mechanisms of education benefits while estimating a comprehensive BCR is an important area of future research.

Another limitation of the study is that we do not adjust the VSL for real income growth over time – including the increase in earnings that results from additional education and the increase that is likely to occur over time for the full population assuming economic growth.

Our BCA for incremental investment in education reflects current recommendations for valuing mortality risk reductions based on available literature, as discussed in Robinson et al. (2019*b*). Those recommendations include both alternative estimates of the population-average VSL and of the use of VSLY estimates to adjust for the age of those affected. More research in LMCs is needed to improve these estimates.

This paper estimates the impact of increasing average years of schooling in LMCs by a year. However, (i) the impact of one additional year at the primary level is likely different from the impact of an additional year at high school or in college; and (ii) the impact of increasing schooling by a year across the different countries in the lower-middle-income group could also be different – the heterogeneous impact of increased schooling while important, is beyond the scope of this study and is another noteworthy topic for future research. Additionally, sensitivity analysis around costs is also out of the scope of the study, and is another area of further research along with additional primary data collection on education costs in low- and middle-income countries.

Further sensitivity analyses could consider other parameter values – for example, varying the impact of female schooling on mortality, or to estimating the impact of schooling on both mortality and morbidity. The mortality risk reduction benefits estimated in this study constitute an underestimate of the benefits because the study (i) does not consider the impact of education on decreasing morbidity; (ii) uses the lower bound estimate of the impact of education on mortality; (iii) fails to include stillbirths averted; and (iv) fails to include a broad range of favourable social impact (for example reduced incarceration rates). Additionally, various controls and model specifications could change the regression estimate of the impact of schooling on mortality. Pradhan et al. (2018) perform this sensitivity analysis at the regression level to estimate the impact of education on mortality using different controls and specifications in the literature, and we use the lower bound of the range of those estimates in this case study. As these points in our discussion make clear, more research will, in this case as in others, add to the confidence and generality of our findings. That said, we nonetheless would judge our broad findings robust and in general conservative.

## Footnotes

1 As defined by the World Bank.

2 Please note that we are assuming a social rate of time preference of zero and an elasticity of the marginal utility of consumption of 2.

## Supplementary material

For supplementary material accompanying this paper, please visit https://doi.org/10.1017/bca.2019.5.

## Appendix

**Table A1:** Data sources used in the note.

Variable	Description	Data sources (see reference list)
Educational attainment (mean years of schooling)	Mean years of total schooling among the population aged 25C. Both overall and sex-specific estimates were used.	Barro and Lee data set, version 2.0
Under-five mortality (5q0)	Probability of dying between birth and exact age 5 at current age-specific mortality rates, per thousand.	UN World Population Prospects, 2017 revision
Adult mortality (45q15)	Probability of dying before exact age 60 for those alive at age 15 calculated at current age-specific mortality rates, per thousand. Both overall and sex-specific estimates were used.	UN World Population Prospects, 2017 revision
Fertility	Total fertility rate (children per woman).	UN World Population Prospects, 2017 revision
Remaining life expectancy at age (a) (L(a))	Average number of years lived subsequent to age (a) by those reaching age (a)	UN World Population Prospects, 2017 revision
GNI per capita (PPP)	GNI per capita, PPP (current international $)	World Development Indicators, 2017
Age-earnings profile	Age-specific wage return of each level of schooling	(Psacharopoulos et al., 2017)
Direct cost of schooling	Direct cost of schooling of one pupil per year	(The International Commission on Financing Global Education Opportunity 2016)
